# The Marion County (Fla.) Medical Society

**Published:** 1896-01

**Authors:** R. P. Izlar

**Affiliations:** Secretary; Ocala, Fla.


### THE MARION COUNTY (FLA.) MEDICAL SOCIETY.

OCALA, FLA., December 10, 1895.

The annual meeting of this society was held yesterday in the office of Dr. R. P. Izlar. There were present Dr. W. R. O. Veal, of Cotton Plant; Dr. D. A. Smith, of Anthony; Dr. S. H. Blitch, of Blitchton; Dr. T. N. Lewis, of Kissimmee; and Drs. Newsom, Hood, Thompson, Smith, Moody, and Izlar, of Ocala.

The following officers were elected for the ensuing year: President, Dr. W. V. Newsom; Treasurer, Dr. W. R. O. Veal; First Vice-President, Dr. D. A. Smith; Second Vice-President, Dr. T. J. Myers; Secretary, Dr. R. P. Izlar.

Various papers were read and discussed; among others, one by Dr. Newsom on "Appendicitis" and one by Dr. Blitch on "Colitis."

On motion, the following resolutions were adopted:

### LIFE INSURANCE EXAMINATIONS.

WHEREAS, Some life insurance companies throughout the United States have reduced the fee for medical examination from $5 to $3.

*Resolved*, That the members of the Marion County Medical Society refuse to make such examinations for less than $5.

*Resolved*, That this preamble and resolutions be spread upon the minutes of the society and published in the medical press of the United States.

Also the following, which was carried:

### DEPARTMENT OF PUBLIC HEALTH.

WHEREAS, This society believes that a Department of Public Health, under control of a Secretary, who should be a member of the cabinet of the President of the United States, is necessary to the welfare of this country; therefore be it

*Resolved*, That this society indorses the bill now pending to establish such Public Health Department, and requests our members of congress to support said bill and aid in its passage.

*Resolved*, That the secretary be requested to transmit a copy of the foregoing preamble and resolutions to our senators and representatives in congress.

*Resolved*, That this preamble and resolutions be spread upon the minutes of the society.

Dr. W. V. Newsom, as President, delivered his annual address to the society on the "History of Medicine."

The members and invited guests afterward assembled in the parlors of the Montezuma, and dinner was served in the dining-room of that hotel.

R. P. IZLAR, M.D., Secretary.

